# Local knowledge about a newly reintroduced, rapidly spreading species (Eurasian beaver) and perception of its impact on ecosystem services

**DOI:** 10.1371/journal.pone.0233506

**Published:** 2020-05-21

**Authors:** Viktor Ulicsni, Dániel Babai, Erika Juhász, Zsolt Molnár, Marianna Biró

**Affiliations:** 1 Institute of Ecology and Botany, MTA Centre for Ecological Research, Vácrátót, Hungary; 2 GINOP Sustainable Ecosystems Group, MTA Centre for Ecological Research, Tihany, Hungary; 3 Institute of Ethnology, MTA Research Centre for the Humanities, Budapest, Hungary; 4 Department of Plant Systematics, Eötvös Loránd University, Ecology and Theoretical Biology, Budapest, Hungary; University of Waikato, NEW ZEALAND

## Abstract

Conflicts caused by reintroduced native species are increasing; however, there is a knowledge gap concerning ecological knowledge and perception of local community members regarding the impact of these species on local ecosystem services and livelihoods. We studied local knowledge about beavers and the perception of their impact on ecosystem services and local livelihoods, and the perception of their general harmfulness and usefulness in Hungary and Romania in three ecologically distinct, diverse rural landscapes. Structured interviews were carried out with 45 knowledgeable and 45 randomly selected local informants. We found that locals were knowledgeable about legal status, biology and behavior of beavers and their diverse impact on nature and ecosystem services. Perceptions included mostly negative impacts on provisioning services, while both negative and positive impacts on regulating and cultural services were perceived, including some contradictory impacts of the species. In spite of the actual and anticipated potential future harms caused by beavers, most people appreciated its precise building mastery and ‘cute’ nature. We argue that communication between nature conservationists and locals should reflect this complexity of perceptions, while reciprocal learning could help to moderate local conflicts and develop adaptive management strategies.

## Introduction

Ecosystem engineer species can contribute considerably to local biodiversity by transforming their environment [1, 2, 3, 4, 5]. This impact is, however, conflicting in many cases. While ecosystem engineer species provide habitats for certain species, they are disadvantageous for others [6, 3]. Both the Canadian and Eurasian beaver (*Castor canadensis* and *Castor fiber*) are good examples of ecosystem engineer species [2, 7, 8]. The role of beavers in watershed restoration was extensively investigated in the case of the Canadian beaver which performs a significant ‘construction activity’ [9, 10].

The Eurasian beaver disappeared from most parts of Europe in the second half of the 19^th^ century [11]. Consequently, their activity in modifying water courses and contribution to biodiversity also disappeared. In Europe, the main purpose of reintroduction was to restore beaver populations, and contrary to the U.S. conservation management, watershed restoration was rarely listed among the specific aims of beaver reintroduction projects [12, 11]. Beavers have mostly recolonized their former distribution range [13, 11, 14, 15, 16]. Although the Eurasian beaver is a native species in Central Europe, and in some areas it spreads rapidly just like that of the golden jackal (*Canis aureus*) in Southeast and Central Europe [17]. As a result, a conflicting situation has unfolded: beavers are protected and their reintroduction is regarded as a success story by conservationists [18], while their activity is becoming a source of conflict between locals and nature conservationists [19]. However, conservationists often fail to consider the diversity of local perceptions [20, 21].

There are several knowledge gaps concerning the social factors affecting local perceptions and human-wildlife coexistence. One example is the relation between the actual harm caused by wild animals and their local perception [22]. Local perception can also be influenced by local ecological knowledge about the given species [23, 24]. Despite the fact that European beavers play a significant role in forming the floodplain ecosystem services, the local perception of its impacts is understudied. Studies about the local perception of the impact of beavers’ activity on ecosystem services are mostly limited to *Castor canadensis*. The main conclusions of these studies are that local perception primarily refers to provisioning ecosystem services [25] and that the attitude of locals could be changed considerably by defining the actual economic loss [19].

Local knowledge, perception and attitude related to wild animals’ activity has important policy and nature conservation relevance [26, 27, 28, 29]. There is, however, a considerable knowledge gap concerning the Eurasian beavers’ impacts on ecosystem services, local perception of the beavers and local attitudes towards them [cf. 30, 31, 32]. A better understanding of local perceptions could play an important role both in nature conservation and sustainable beaver management, for example, by promoting the efficiency of social learning and resolving or avoiding further conflicts [22, 27]. This has been shown by several studies about the local perception of and knowledge on *Castor canadensis* [33, 34] and about the attitude of locals towards the species [35, 36, 37].

Because of the complexity of the conflicts regarding the beaver’s activity and its dependence on local social and environmental contexts, it is necessary to conduct policy-relevant interdisciplinary research in multiple, distinct landscapes [27, see also 19].

Our main objective was to study the local knowledge and perception of the European beaver’s impact on local ecosystem services in ecologically diverse rural landscapes at three locations in two different countries. Namely, we wanted to explore:

- local knowledge about the Eurasian beaver (e.g. protection status, reintroduction history, local distribution, feeding habits);
- the local perception of negative or positive impacts of beavers on local provisioning, regulating and cultural ecosystem services and on nature and local livelihoods in general.
- The local perceptions on the harmfulness and usefulness of beavers and its impacts on nature and the lives of locals.

We also compared knowledge and perceptions held by knowledgeable and randomly chosen informants in the three studied landscapes.

## Study area and methods

### Studied regions

Three regions in Central Europe were chosen where beavers are present in waters close to settlements and where their activity affects the floodplains considerably. The study areas are in different types of watersheds (streams, small rivers and river branches of large rivers) and the local communities are in different socio-economic situations (traditionally farming and partially modernized).

**Fig 1:**
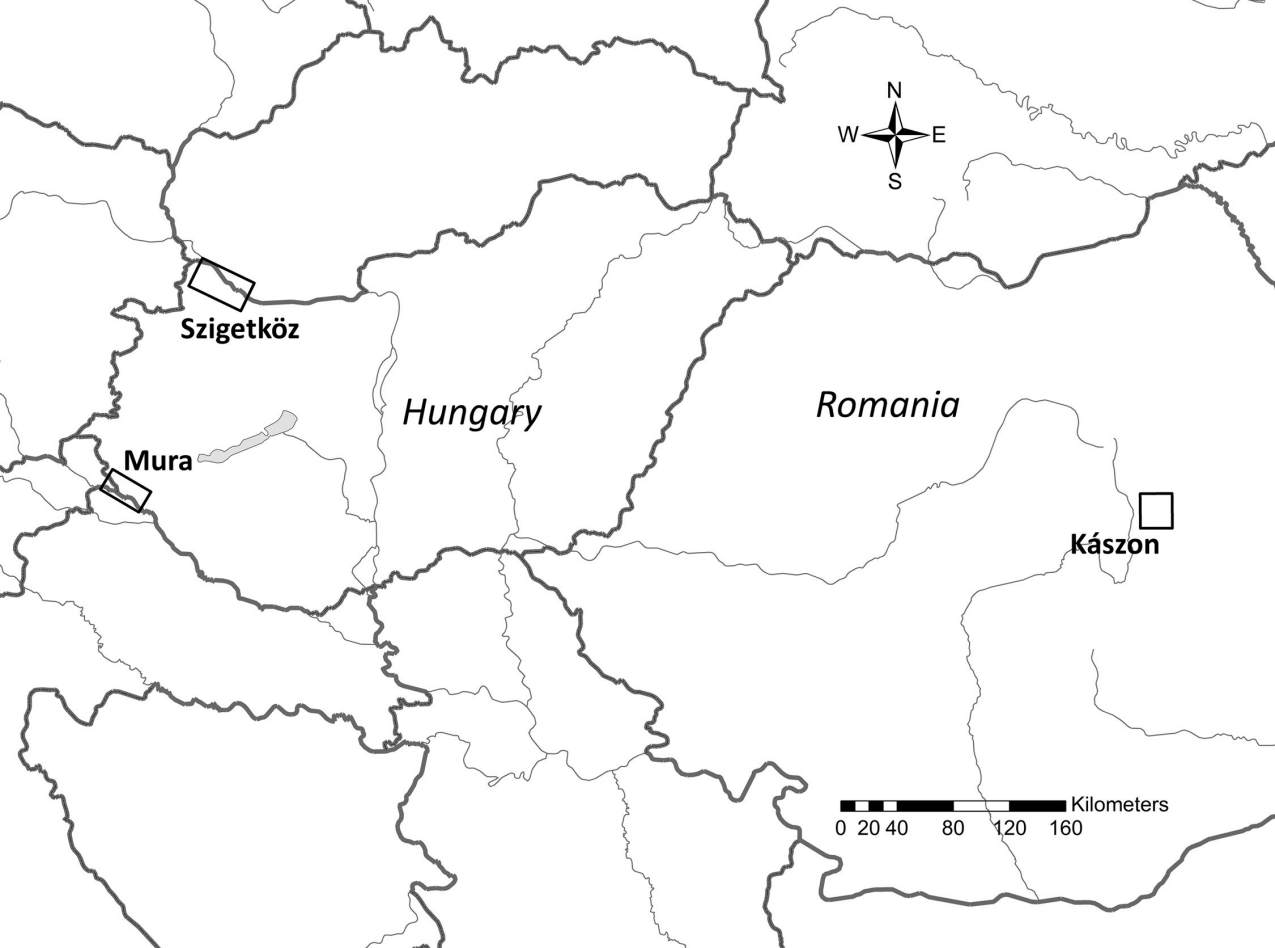
Map of the study areas in the Carpathian Basin, Central Europe. Rectangles indicate localities of data collections. Country borders: thick grey lines, main rivers: thin grey lines (source: Natural Earth). Source of base map: Arc.GIS.10.1 (ESRI).

Studies were carried out in the Kászon Basin (Romania), in the Szigetköz (Hungary) and in the Mura River valley (Hungary) (Fig 1). The three areas where the data were collected, had 2100, 1600 and 1200 inhabitants respectively in neighbouring villages in every landscapes.]. In the Kászon study site, smaller streams are accompanied by small-scale hay meadows, *Salix fragilis*, *Salix purpurea* and *Alnus incana* forests, and settlements. About 60% of the population makes a living out of small-scale farming [38]. Beavers build dams which impound the water of the streams. Along the Mura River, the hay meadows abandoned in the 1950s are occupied today by *Salix alba*, *S*. *fragilis* and *Populus alba* forests. On the large-scale arable fields often extending to riverbanks, the most important crop is maize, and about 15% of the population makes a living out of agriculture [39]. The area was designated as protected in 2007 [40]. The Szigetköz study site is entwined by river branches of the Danube River. Mixed *Salix alba*, *S*. *fragilis*, *Populus* x *canescens* and *P*. *nigra* forests and *Populus* x *canadensis* plantations extending to the river banks are typical. Only a very small ratio of the population lives off of agriculture [41]. The landscape was considerably transformed by the river regulations and the Bős-Nagymaros hydroelectric dam. The area has been protected since 1987, and it is an important tourist destination [40].

### The beaver in the study areas

As a result of overexploitation, the Eurasian beaver went extinct in the Carpathian Basin in the 19^th^ century (the last record in Romania: 1824 [42], in Hungary: 1865 [43]). The species has spontaneously recolonized all the three study sites from nearby (less than 100 km) areas where they were reintroduced. Beavers appeared in Kászon around 2009 (introduced between 1998 and 2001 along the Olt river [42]). In the Szigetköz, beavers were first recorded in 1985–86 (recolonizing from Austria). They appeared in 1997 along the Mura River (A. Lelkes—personal communication, January 22, 2017). Reintroduction was carried out in 1997 in neighboring Croatia along the Dráva river [44]. The Eurasian beaver is protected in both countries. In Hungary at the time of the interviewing, legislation did not allow the control of beaver populations [45]. From 2016 on, removal permits for a given number of beavers were granted. There is no data on the size of the beaver population in Kászon. Beavers occur in most streams but the population is presumably under one hundred individuals. In the Mura region there were 35–45 families present in the studied period [46]. It was estimated that there were 352 families in the Szigetköz in 2017 [47].

### Methods of data collection

Between 2014 and 2016, structured interviews were conducted with 30 local people for each of the three study sites in the following settlements: Plăiesii de Sus, Plăiesii de Jos, Casinu Nou, Imper, Iacobeni (Kászon); Dunasziget, Kisbodak, Ásványráró (Szigetköz); Kerkaszentkirály, Muraszemenye and Murarátka (Mura). Half of the informants (altogether 45 people) were recommended by residents (with snowball method according to Biernacki and Waldorf [48]; Heckathorn [49]) and local community leaders as ‘inhabitants knowledgeable about beavers’ (called knowledgeable local informants–KLI). They were expected to have more extensive knowledge about beavers than the general public and more personal experience as well. The sampling was considered representative as the number of new memes was saturated (S1 Fig). Comments like “they slow down water” or “I like them” were considered as one meme (information unit, see also S1 Table). The other half of the informants provided additional information and helped in representing the average population’s knowledge. They were chosen randomly (randomly-selected local informants–RLI), without any special recommendation (met on the street, knocked at a random house, etc.). These people could be either under-informed or knowledgeable regarding beavers. To avoid the distortion resulting from the different methodologies we used the same method for interviewing RLIs as for KLIs. The average age of the 90 informants was 51 years (Kászon: 45, Szigetköz: 54, Mura: 55), and 18 informants were female. The initials (K, S, and M) after KLI and RLI refer to the study areas Kászon, Szigetköz, and Mura, respectively.

The majority of the interviews were recorded with a voice recorder and then transcribed (altogether 73 hours and 42 minutes of recordings with an average length of 49 minutes per interview (Kászon: 48, Szigetköz: 53, Mura: 45). Prior informed oral consent was obtained before all the interviews, and ethical guidelines suggested by the International Society of Ethnobiology (ISE 2006) were followed. The data were collected and analysed anonymously.

Twelve scientific and conservation experts (SCE) from Hungary and 6 from Romania were also interviewed in the same time period, using the same interview sheet, to improve our understanding about the local situations. Neither detailed analysis of the SCE interviews nor direct comparison of their content with the local peoples’ interviews were objectives of this study. In our experience, Hungarian, Romanian, and European literature in general was not enough to keep pace with changes in the local beaver situations and these expert answers helped considerably in the discussion of our results.

The interview sheet included both closed and open-ended questions (S1 Data). Questions were phrased and selected to allow both quantitative and qualitative analysis. As most of the informants were not aware of certain scientific concepts (e.g. ‘ecosystem services’ or ‘biodiversity’), we needed to change questions with ecological terminology to questions with basic terms in the interview sheet.

### Methods of data analysis

The answers to the questions eliciting encyclopaedic knowledge (protection and hunting status, time of introduction, local distribution, predation, dangerousness, feeding habit, population dynamics) were summarized based on the ratio of respondents giving ‘correct’ answers. To decide which answers were ‘correct,’ we used the scientific literature and answers of the scientific and conservation experts (see in 2.1. and 2.2.) from the two countries.

We used CICES 4.3 classes for ecosystem service categories [50]. The impact of beavers on ecosystem services was analysed by extracting all information from the transcribed texts about each service mentioned by the 90 informants to Microsoft Office Excel data-sheets (partially in S2 Table). The beavers’ negative and positive impacts on provisioning ecosystem services were analysed based on the number of respondents who mentioned certain categories. For regulating and cultural ecosystem services, the total number of informants mentioning them, and the mentioned memes (information units) were counted. As it was mentioned above, one simple statement was considered as one information unit, one meme. The informants’ personal involvement (direct effect on informants’ property) was also estimated by defining the number of the personally (directly) affected informants.

Overall perception of beavers’ usefulness and harmfulness was elicited using multiple-choice questions (“Are beavers useful or harmful?”) and also free listing ones (“What kind of benefits could you mention?”) and a 3-grade scale (negative, -1; neutral, 0; positive, +1, e.g. “What impact do the beavers have on your life?”) (S1 Data). Both the overall perception of beavers’ usefulness and harmfulness and the informants’ personal involvement were analysed by the number of respondents and the number of mentioned memes. We counted number of respondents based on number of informants giving answer or ‘I don’t know’ in the interviews. Statistical analyses (basic statistics, mean and relative frequency calculation) and figures were constructed in Microsoft Office Excel program.

## Results

### Local knowledge about some key features of European beavers

Locals had a deep and detailed knowledge on beavers’ legal status, biology and behaviour in all three studied areas. The protected status of beavers was known to 95% of the respondents (95% of both RLI and KLI) (S2 Fig). The RLI-K group knew much less about the shooting of beavers (45%) than any of the other 5 groups (80–100%). The method of reintroduction was moderately known by both the RLI and KLI groups. All informants in the Mura and Szigetköz study areas perceived a trend of population growth, and nobody indicated population decrease.

Sixty-two percent of the respondents were of the opinion that beavers have no natural enemies today (RLI and KLI groups: 67% and 57%, respectively). In some cases, wolves (*Canis lupus*), bears (*Ursus arctos*) and stray dogs (especially in Kászon) as well as humans were mentioned as predators. Ninety-eight percent of respondents did not consider beavers to be a threat to humans. A higher ratio of informants in the KLI groups (compared to the RLI group) thought that beavers were exclusively herbivores. The proportion of respondents supposing beavers to be exclusively herbivores was the lowest in Kászon (38%). Two-thirds of the informants knew which parts of trees are used by beavers.

The date of local reappearance of beavers and how far they would go from water bodies were known to 79% and 84% of the respondents, respectively, with no considerable difference between the RLI and KLI groups (S3 Fig). Concerning the date of reappearance, the highest deviation was found in the Szigetköz region.

### Perceptions of the impact of beavers on ecosystem services

#### Provisioning services

**Fig 2:**
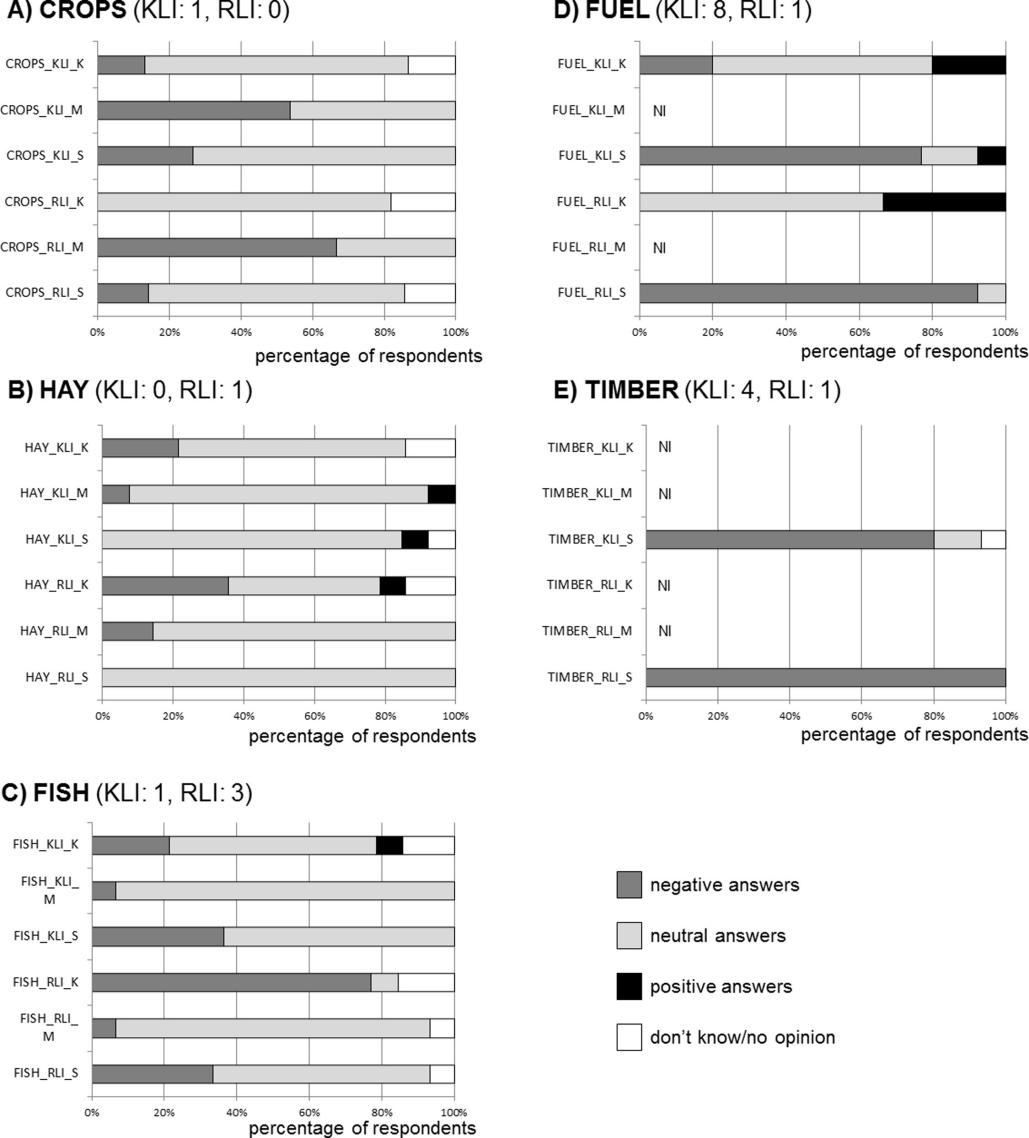
Perceived impacts of beavers on provisioning ecosystem services based on using CICES 4.3 classes (KLI: Knowledgeable local informants, RLI: Randomly-selected local informants). First axes show percentage values of perceived negative, neutral and positive impacts. (negative, -1; neutral, 0; positive, +1). PAI: numbers of personally-affected informants in the given service class. NI: not interpreted as either there is no timber production along rivers (Kászon) or the riverine forests are protected and thus fuel or timber cannot be exploited (Mura).

The impact of beavers on provisioning ecosystem services was perceived as negative or neutral (Fig 2). Positive impacts were perceived only in 9 cases. Damage to crops was perceived as negative or neutral. Both the RLI and KLI groups were of a similar opinion. Informants were aware of the damage in all three regions (Mura: feeding on maize and using it as building material, Szigetköz: feeding on maize, peas, sunflowers, sugar beets, cereals and fruit trees, Kászon: feeding on mangel beets), but considerable loss of their own crops was not mentioned. Only 2% of informants experienced loss of their own crops.

The beavers’ impact on hay was perceived as slightly negative in Kászon. The impact of the Eurasian beaver on fish as a provisioning service was perceived as negative or neutral in all three regions. The worst opinion was formed in Kászon. In the other two regions, informants noted that beavers disturbed fish and their movement hindered access to the service and interfered with fishing.

The perception of the impact on fuel and timber wood was outstandingly negative in both groups of the Szigetköz region. As for timber, the damage in poplar plantations was regarded as almost exclusively negative in Szigetköz area (100% RLI-S, 86% KLI-S respondents). The beavers’ impact on fuel wood was considered slightly positive in Kászon: “*They* [the poor people] *take home the wood that the beaver has felled*, *and so have fuel for winter*.” In the Mura region, the impact on timber extraction was not relevant as it is forbidden by law in the studied area. “*It is a protected area*. *Felling is not allowed*. *After the tree falls*, *it remains there to decompose*.”

#### Regulating services

**Fig 3:**
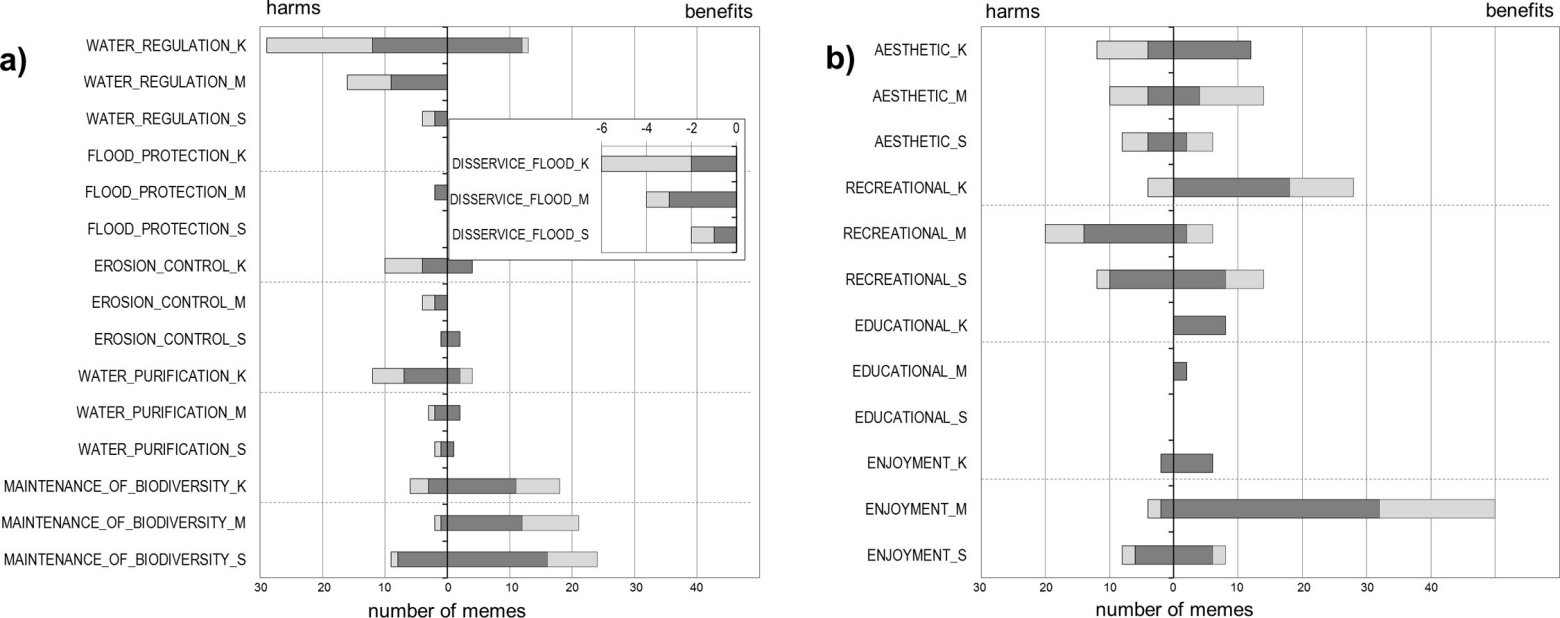
Perceived impacts of beavers on regulating (a) and cultural (b) ecosystem services (in three regions: K: Kászon, M: Mura, S: Szigetköz). First axes show the total number of memes mentioned by the 90 informants. Dark grey: knowledgeable local informants (KLI), light grey: randomly-selected local informants (RLI). Inserted graph shows perceived ecosystem disservices on flood protection infrastructure in the three regions.

Impacts on regulating services were perceived as both positive and negative (Fig 3A). Almost everybody (25 informants) in Kászon mentioned the beavers’ negative impact on water regulation, but positive impacts were also recognized (e.g. the level of ground water rises, the water regime is more balanced, floods are moderated and water is available even in drought). Little information was mentioned on beavers’ impact on flood protection but flood risk as an ecosystem disservice caused by beavers’ activity was mentioned several times (2 and 12 memes, respectively). In Kászon, beaver dams swept away by floods were mentioned as a potential flood risk for villages. On the other two sites, the damage to man-made dykes, especially burrows, was mentioned. The trees cut by beavers may also get caught, for example by bridges, and cause flooding.

The impact of beavers on erosion control was mentioned by only a few informants and their opinion was mostly negative: “*the banks become unstable*, *water cuts in* [after trees were removed by beavers],” “*river beds become deeper*,” “*they burrow the banks*, *make dens; water can cut in more easily and deeply*.” On the other hand, some informants of the KLI groups mentioned positive impacts as well: “*they are beneficial because they slow down the flow of the water and so water becomes less erosive*,” “*they stop riverside erosion* [by felling trees and steering water away from the banks].”

Beavers’ impact on water purification and water quality regulation was also mentioned several times, especially in Kászon. It is mostly considered negative: “*water slows down*, *gets warmer and poorer in oxygen*,” “*water is full of decomposing plants*,” “*the stream is befouled* [by having all kinds of things stuck], ” “*branches and logs are floating*,” and “*mud is stirred up*.” “*Filtering water*” was mentioned as a positive effect.

Maintenance of life cycles and conservation of habitat and genetic diversity was mentioned by the same proportion of informants in both the RLI and KLI groups. Almost two-thirds of the informants (62%) brought it up and most of them referred to it as positive. KLI groups mentioned more memes than RLI groups. A few people declared that hay meadows are spoiled by flooding caused by beavers (as the proportion of sedges rises), but most of the informants pointed out the increase in biodiversity (more amphibians, fish, aquatic birds, new plant species). In some cases, beavers’ positive role in the ecosystem was not even questioned as it is a native species (“*they do have a role in nature*”). KLIs mentioned positive and negative impacts in similar numbers, 55 and 63 memes, respectively, while RLIs perceived more negative impacts (50) and only 25 positive ones.

#### Cultural services

Impacts of beavers on cultural ecosystem services were mostly perceived as positive (Fig 3B). Regarding aesthetic services both positive and negative impacts were mentioned. Some people reported that the landscape looks unkempt: “*it becomes chaotic*, *messy*” and “*untidy*.” On the other hand, there were positive comments as well: “*beavers clean the banks*” (i.e. they gnaw off weeds). Beavers are mostly perceived to affect recreation (fishing, tourism) positively in Kászon, and negatively in Mura. In Szigetköz answers were more balanced. Beavers’ impact on environmental education and awareness was rarely mentioned (K: 4, M: 1), but when it was, the comments were always positive (*“camps could be organized to show them to children”*). Informants (most often in the Mura region, 58%) reported that they liked beavers and admired their activity (they are cute, build dams and lodges, work with mechanical precision, their gnawing is perfect): “*It’s nice to encounter them*, *I like them very much*.”, “*They have such cute faces*. *You just can’t dislike them*.” The feelings were sometimes ambivalent: “*It’s good to have them*, *but there are just too many of them*.” “*They give me half a day of extra work each year*, *but that’s all*. *I can live with that… I enjoy having them here*.”

### Usefulness-harmfulness and overall impact of beavers

**Fig 4:**
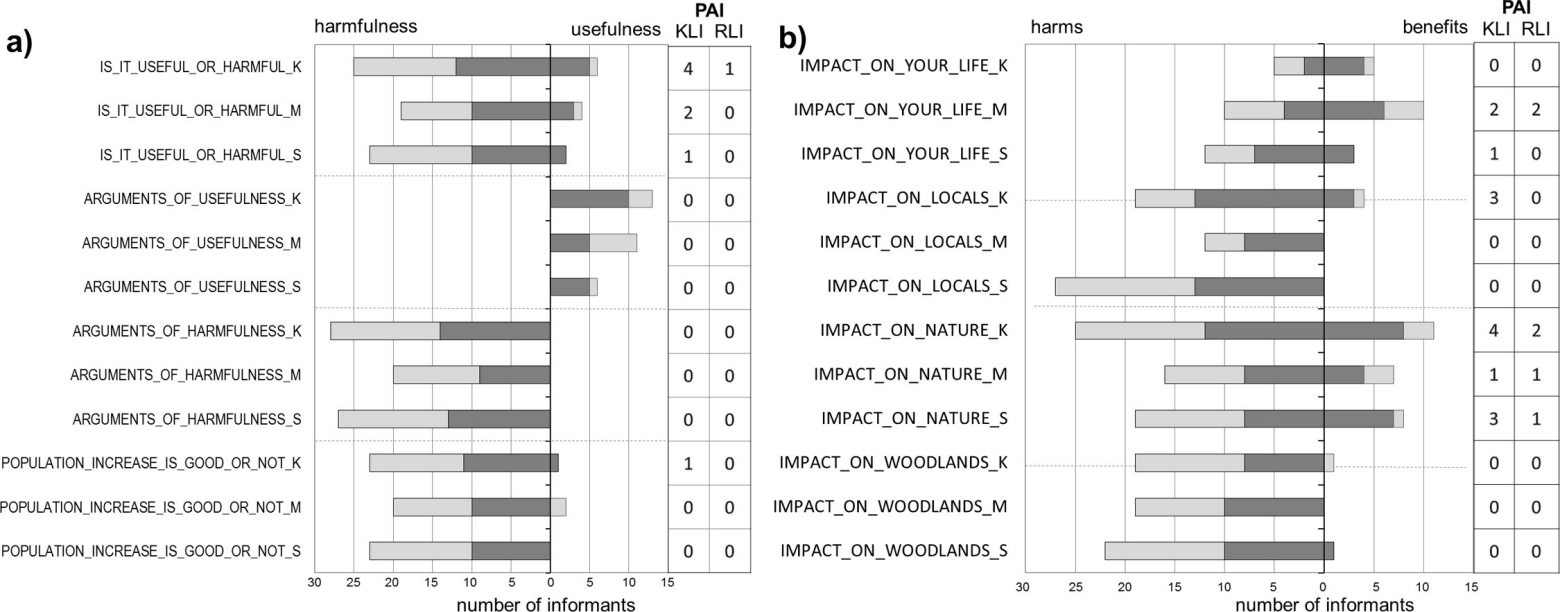
**a** Perceptions of beavers by local informants considering questions on usefulness and harmfulness of beavers (K: Kászon, M: Mura, S: Szigetköz): ‘Are beavers useful or harmful?’; ‘What kind of benefits could you mention?’; ‘What kind of harms could you mention?’; Is this population trend good or bad in your opinion?’; **b** ‘What impact do the beavers have on nature in general / on riparian woodlands / on your life / on other inhabitants of your village?’. Dark grey: knowledgeable local informants (KLI), light grey: randomly-selected local informants (RLI). The numbers in the last two columns are the number of informants giving both positive and negative perceptions.

Answering the multiple-choice question about usefulness and harmfulness (‘Is the beaver useful or harmful?’), most respondents (72%) regarded beavers as harmful and only 5% as beneficial (Fig 4A). However, ambivalent perceptions were also common: eight informants (9%, K:5, M:2, S:1) thought them to be both harmful and useful (“*partly good and partly bad*”). When usefulness was explicitly asked about, there were surprisingly many positive answers. In Kászon, 46% of the informants acknowledged the beaver to be useful in some ways in the free listing questions but only half of them gave a positive answer to the previously asked question. Altogether 75 memes referred to harmfulness while only 30 memes to usefulness.

Growth of the local beaver population was considered negative in all three sites (Fig 4A). Even those who liked beavers thought that there were too many of them: “*I’m glad that they are here*, *but there’s more than enough*.”, “*If their population grows we will be in trouble*.” There were a few accepting opinions as well (but only from 7 informants): “*If they are here*, *let them be*!”, “*I’m an animal lover*, *it’s just natural that their population increases*, *I don’t mind that*.”

Beavers’ impact on nature was perceived as negative by 57% of the respondents (Fig 4B) but positive things were also mentioned in all three sites (by ca. 30% of respondents). Regarding forests, both groups in all three sites perceived beavers’ activity as negative but also noted that it’s not really important: “*It’s a jungle anyway*.” “*It doesn’t make any difference*.”, “*there aren’t fewer trees because of them*.” In Kászon, negative impacts on hay meadows were mentioned but also several positives.

As far as the beavers’ impact on personal life is concerned, half of the informants (44 informants) declared that beavers don’t bother them (Fig 4B). Two people noted that beavers do not affect them but they were annoyed anyway. Negative perceptions mostly referred to damage to provisioning services. In Kászon it was exclusively the hay (5), in Szigetköz timber and fuel wood (9), and fish by the Mura (2).

The questions about informants’ personal relationship with beavers revealed many indications of positive attitudes: “*one more curiosity*,” “*it is nice to see a beaver lodge*, *what a complex building*,” “*it’s a joy for children to observe*,” “*they are beautiful*,” “*I love them*.” Five informants mentioned both positive and negative relationships. Four of them were from Mura: “*They give me a lot of work*, *but as I’ve said I like animals*. *I can live with them even though I have a little bit more work to do because of them*,” “*If they didn’t gnaw my thujas*, *I wouldn’t mind them*. *I like animals; it’s nice to see them*.”

As for the relationship of the local community with the beavers, mostly negative opinions were expressed (11 informants) and hardly any positive ones. In most cases there was no explanation: “*they don’t like them much* [in the village].” In Kászon the damage to hay meadows was mentioned again (by 7 people). Some answers were ambivalent: “*both good and bad*,” “*in some parts even properties are threatened*, […but] *wood* [that was felled by the beaver] *can be used as fuel*, *that’s good*.” The positive impacts of beavers on the villagers’ lives were only expressed in Kászon: water regulation, state of nature and beauty of nature: “*God has created beavers with a purpose*.” To the ‘Who benefits from the presence of beavers?’ question, the most abundant answer was “*no one*” (45%). Informants also declared that they are good for the nature conservationists and tourists (35% of respondents) and only rarely to locals (8%). Analysis of all the answers in Fig 4 shows that 71% of positive memes were mentioned by KLI group members (67 out of 95), while they contributed only 49% of the negative memes (202 out of 413).

## Discussion

### Local knowledge about key features of beavers

Considerable local knowledge about beavers was found in all three study sites, even though in some places beavers only reappeared 5–10 years ago. Similarly, the Canadian beaver is also a well-known and salient species all through its natural range even in places where they have just recently appeared ([25, 19]. The time and method of beavers’ local reappearance [cf. 18, 49, information from SCEs) were well estimated by both KLIs and RLIs despite the fact that they knew little about the actual reintroduction methods. Local knowledge about the conservation status and hunting status of beavers reflected the differences between Hungarian and Romanian regulations [38]. In Romania, regulations allow even protected species to be conditionally shot or trapped [51, 52].

The species’ local distribution was well known and local knowledge was consistent with the scientific literature [53, 47] and the experiences of the SCE group. Scientific literature [53, 47] and information from SCEs confirmed these local perceptions. In Kászon and by the Mura the beaver population is still growing. In the Szigetköz, the population has probably stagnated, but locals still perceive growth. Local knowledge about the biology of beavers was also consistent with the literature and SCE information regarding both distance reached from waterbodies [44, 54] and predators [55].

The use of woody plant species by the beavers was well known to locals as far as species were concerned, but they knew less about the plant parts beavers eat. This is probably because it was much easier to discover cut trees than other evidence of feeding (e.g., small gnawed twigs) [cf. 54, 30, 56, 57, experience of SCEs]. In Kászon, locals have presumed a connection between the arrival of beavers and the decline in the abundance of fish. This might have contributed to the false impression of beavers eating fish. Beaver dams were common in Kászon. That is why many locals could have believed that the purpose of felling the trees was dam building rather than feeding. We conclude that beavers were viewed from different perspectives at the three sites but local knowledge about their legal status, biology and behaviour was extensive everywhere.

### Impact of beavers on ecosystem services

#### Provisioning services

Consistent with the literature and SCE opinions, both groups at all three sites evaluated damage to cultivated crops realistically [cf. 30]. By the Mura, wild boars were considered to be more harmful than beavers. Damage to crops is rarely reported in literature [e.g. 34]. The impact of beavers on provisioning services could be related to the human use of the immediate waterfronts. In Kászon the streams are accompanied by trees and hay meadows, by the Mura arable fields extend to the river in a dominantly forested landscape, and in the Szigetköz there are extensive plantations on the riversides. According to Caballero-Serrano et al. [23], it is typical that the value of damage to provisioning services is estimated by the various local stakeholder groups differently than by biologists and conservationists. Our results indicate that these estimates can even differ from region to region. It was conspicuous that in several cases, negative impacts mentioned were just potential impacts, and the beavers’ landscape-forming activity was perceived as negative regardless of its actual economic impact. For example, species (like *Salix* spp.) mentioned as damage to fuel wood are not of high commercial value.

Perception of beavers’ impact on fish was contradictory, as reported in the scientific studies [cf. 1, 6]. Informants mentioned that beaver dams moderate the flow of water and it becomes disadvantageous for some species, like trout [c.f. 11]. On the other hand, informants also mentioned that this is beneficial for species that prefer slow-flowing water [c.f. 58]. Similarly, it was mentioned that the trees falling into the water may provide shelter and spawning grounds for several fish species [c.f. 59]. Some of the informants, especially in the Szigetköz, declared that beavers have a negative impact on fish without mentioning any specific reason, most likely because of their general dislike of the animal. We suppose that local opinion was influenced by the number of fish caught [see also 60] rather than conservational aspects like fish diversity or abundance of rare fish species.

In the Szigetköz there are extensive poplar plantations right by the river [61] and informants reported considerable damage to them (especially RLIs, because they are less aware of specific aspects of the impacts and usually provided more general answers). Similar damage to timber stock has been reported from North America [36, 34, 37]. In the Szigetköz people regularly gather fuelwood from the floodplains, and damage was mentioned by several informants. We could not evaluate the impact on timber and fuel services in Kászon, as everywhere in Romania, there is no timber production along small streams [30] and by the Mura River the riverine forests are protected and thus cannot be exploited.

#### Regulating services

Impacts on regulating services were mentioned mostly as negative and provided important arguments for regarding beavers as a ‘nuisance’ [cf. 37, 30, 62, 63]. Flood risk as a disservice is a serious beaver impact in the US as well [37, 34, 60]. In Kászon the impact of beaver dams and impoundments was said to be substantial. Based on SCEs’ information, in Romania there were a few occasions when the increased flood levels caused by beaver dams endangered villages. Along the big rivers of Hungary, the actual problem was the burrowing of dykes and embankments. In contrast, damage to the irrigation system, typical in North America [34], has not been mentioned in the investigated areas. The regions around the world where the impact of beavers is studied vary considerably in amount of precipitation, land use, human population and geomorphology. This variability of landscape characteristics may be partly responsible for our limited understanding of how much beavers influence ecological systems [27].

As for erosion control, the literature clearly indicates positive impacts [64, 65, 61], while locals mostly perceived negative ones. That may be because they were more conspicuous than the mostly indirect positive impacts. Locals may have perceived beavers’ influence on water purification as negative because their indicators (like decomposing organic matter, floating bits of trees) were different from those of scientists. Müller-Schwarze [66], Czabán [61] and Law et al. [67] argue that the most important impacts of beaver ponds are that they retain sediment, are sinks for nitrogen, and decrease pH to an optimal level (which are not perceivable by locals).

Informants often assessed the impacts on life-cycle maintenance, habitat and genetic diversity differently from the land user’s and from the ecological points of view. Some informants reported undesirable habitat changes related to their own land management problems while others appreciated the creation of new habitats. An unexpectedly high number of informants provided comments with ecological relevance (51%). It is well established that beavers increase the number of species, and contribute to the maintenance and creation of habitats [1, 2, 68, 61, 14, 5].

#### Cultural services

The perception of beavers’ impact on cultural ecosystem services has brought up interesting attitudes. Informants in Kászon considered the “*mess*” and “*untidiness*” [cf. 69] generated by beavers as a problem because locals’ semi-traditional land-use system makes them partial to the ‘tidiness’ of the man-made local landscape. While studying brown bears, Kellert [70] revealed that aesthetic values are important factors affecting attitudes toward wildlife. Land owners in the US benefit from the aesthetic (and thus recreational) value of beaver ponds [33, 34, 35]. In post-communist countries people are often suspicious about any kind of change [30, 71], which might explain the negative perception of beavers’ transformation of the landscape. This fear of change and of possible intensification of impacts and damage is probably expressed in the almost unambiguously negative perception of population growth [cf. 71]. Local communities would prefer the beaver population to stagnate or decrease in other regions of the world as well [34, 35].

The informants, who perceived beavers’ impact on recreation as negative, referred to the disturbance of fishing and aesthetic points [see also 60]. Ambivalence was common: several people mentioned that anglers are annoyed because beavers make fishing difficult (primarily by frightening fish away), others said that beavers didn’t make any difference. An unexpected finding of our study is that locals considered enjoyment (as an ecosystem service) to be one of the most important positive impacts of beavers. Sometimes this made people more forgiving even if beavers were a ‘nuisance’ to them. Others also found that beavers can emotionally engage a broad segment of the public [37, 33, 32, 35, 30, 60], but they do not mention the forgiving attitude. This phenomenon might be explained partly by beavers being a cute animal and a skilful builder, and partly by the persistent positive communication of nature conservationists (B. Bakó, personal communication, March 2, 2018).

### Overall perception of beavers by locals

Perception of the harmfulness and usefulness of beavers was greatly influenced by their marked impact on provisioning services and various smaller ‘nuisances’ (e.g. felling of shade trees, or gnawing of garden plants). Törnblom et al. [27] also point out the contradictory character of the species. While evaluating harms and benefits, both real and potential impacts came up, and informants presumed that beavers influence other people’s lives more negatively than their own. On the other hand, benefits and positive impacts were also mentioned (similarly to the US and Switzerland; Wigley and Garner [37], McKinstry and Anderson [34], Meyer [32]). Even though the species was basically considered a ‘nuisance,’ informants frequently argued that humans, floods and other wild animals (like wild boars, deer, cormorants, and otters) cause more damage than beavers. However, this did not influence the general opinion that beavers are harmful and ‘nuisances.’ In most cases, there was no considerable difference between the perception of the RLI and KLI group members, and the proportion of informants perceiving the positive impacts of beavers was generally higher in the more knowledgeable KLIs.

Locals did not recognize the conservational benefits of beavers in their personal life. The presence of beavers was considered to be beneficial for outsiders (e.g. nature conservationists, animal rights activists, tourists) rather than themselves. In Kászon, traditional land-use practices are still present; people live relatively close to nature. This may be one of the reasons why people there often find something beneficial in the elements of nature, even in beavers.

### Suggestion for better management of an ‘adorable nuisance’

In many East-Central European cultural landscapes there are still extensive semi-natural riverine areas with spontaneously evolving habitats where the presence of beavers could be justified. Beavers are able to transform the ‘order’ of the human-controlled cultural landscape, and that makes them very effective conservational ‘tools’ (ecosystem engineer species). Locals had deep knowledge about beavers and diverse perceptions of beavers’ impacts. Impacts on provisioning and regulating services were perceived mostly as negative, while impacts on cultural ecosystem services were perceived much more positively. Beavers were perceived as a ‘loveable nuisance.’ The lack of local understanding of priorities and objectives of nature conservation is well illustrated by an informant’s remark: “*If beavers are so harmful*, *why are conservationists spreading them*?”

As the beaver situation changes rapidly and dynamically, it is important that beaver management be adaptive [33]. Studies based on interviews can effectively assess the knowledge, perception and attitudes of locals, which are strongly determined by local ecological and social contexts. The impacts of management regulations on beavers and locals and their local perception would be also worth monitoring.

Locals and conservationists often use different indicators to assess certain ecosystem services (like water purification, fish populations) and they view impacts on the local ecosystem services from different perspectives. Their understanding of biodiversity is also different. Communication with knowledgeable locals who are–as our results show—generally more receptive to regulating services could lead to satisfactory compromises and understanding in beaver management. Naturally, it would be beneficial to adjust the content of nature conservation communication materials to the already existing and fundamentally correct knowledge that locals have about beavers.

We argue that strengthening cooperation between nature conservationists and locals could moderate present and potential future conflicts. Two types of conflicts arose that have to be managed separately: conflict between beavers and locals and conflict between locals and conservationists introducing and protecting beavers. In the study areas, the local traditional communities’ ‘instinctive concern’ for the fate of their local natural resources is still pronounced, and in their scenarios the beaver is a disrupting factor. In our opinion, it is important to take this into account when managing conflicts. Cooperation of locals and conservationists could be based on the positive feelings about beavers like cuteness, curiosity, and building mastery.

## Supporting information

S1 Fig Cumulative number of memes mentioned by knowledgeable local informants (KLIs) and randomly-selected local informants (RLIs). (DOCX)

S2 Fig Percentage of correct answers related to the key features of beavers. (DOCX)

S3 Fig a) The moving distance of beavers from bodies of water as perceived by the local informants b) The time of beavers’ local reappearance in years, as perceived by the local informants.
(DOCX)

S1 Data Questions. The data sheet used for the interviews.
(DOCX)

S1 Table Some typical examples of the diverse perceptions of beavers related to regulating and cultural services. (DOCX)

S2 Table Database of ecosystem services related to the Eurasian beaver in Kászon, Mura and Szigetköz. (DOCX)
