# Supplementary material for: Local knowledge about a newly reintroduced, rapidly spreading species (Eurasian beaver) and perception of its impact on ecosystem services
Source: PLoS One. 2020 May 21;15(5):e0233506. doi: 10.1371/journal.pone.0233506 (PMC7241770; doi:10.1371/journal.pone.0233506)

**S1 Fig.** Cumulative number of memes mentioned by knowledgeable local informants (KLIs) and randomly-selected local informants (RLIs) in a) Kászon, b) Mura, c) Szigetköz.
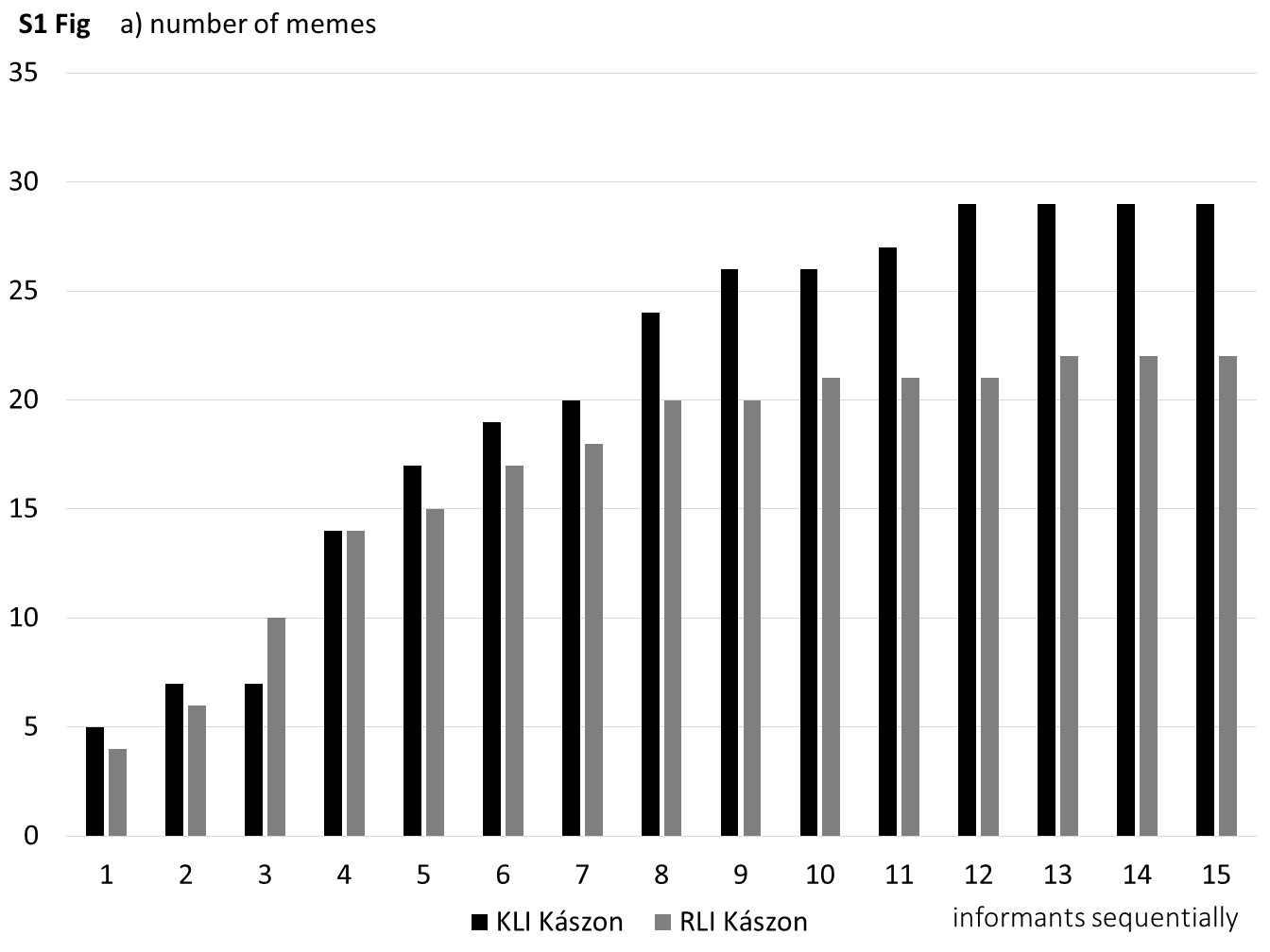

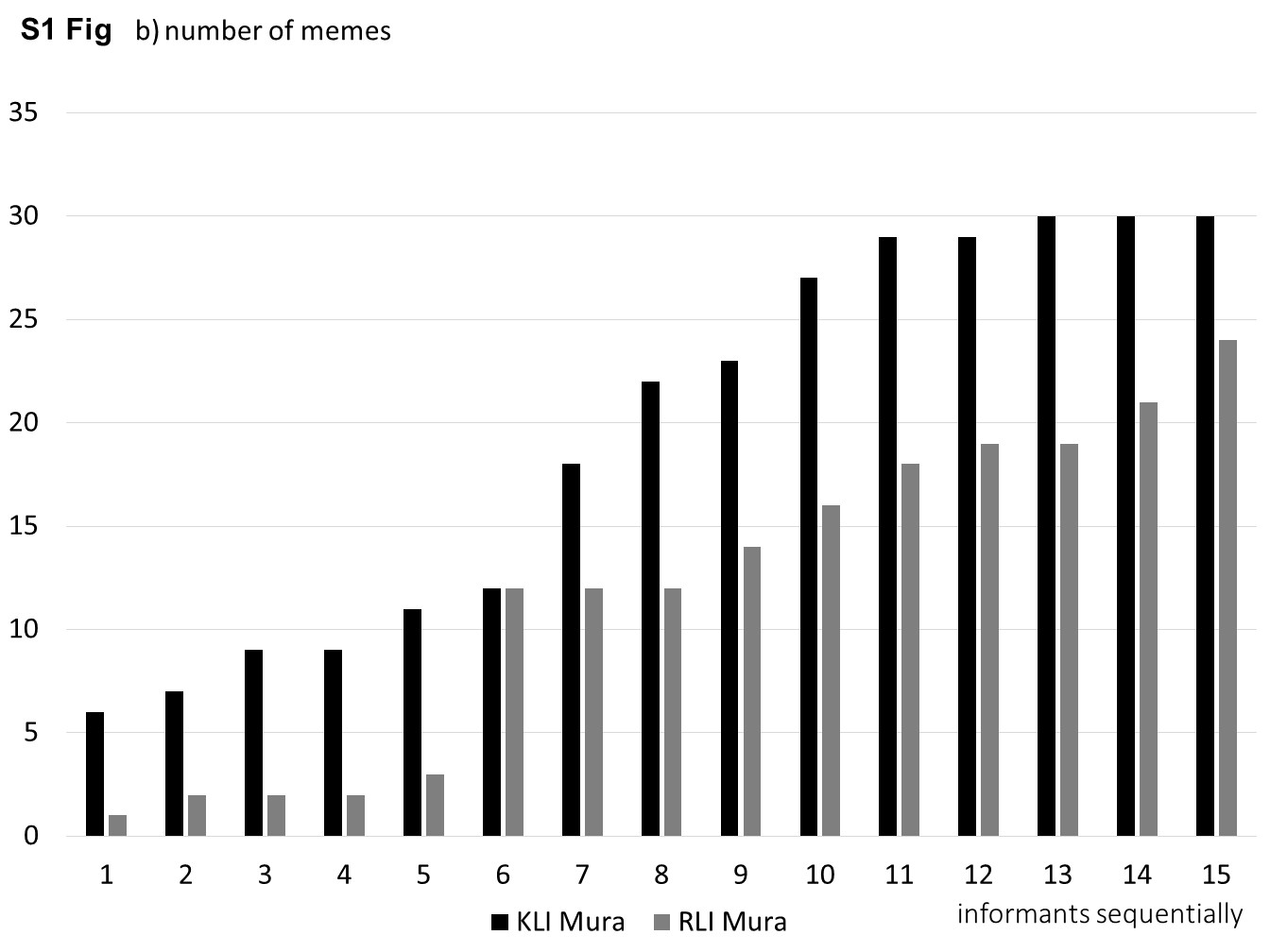


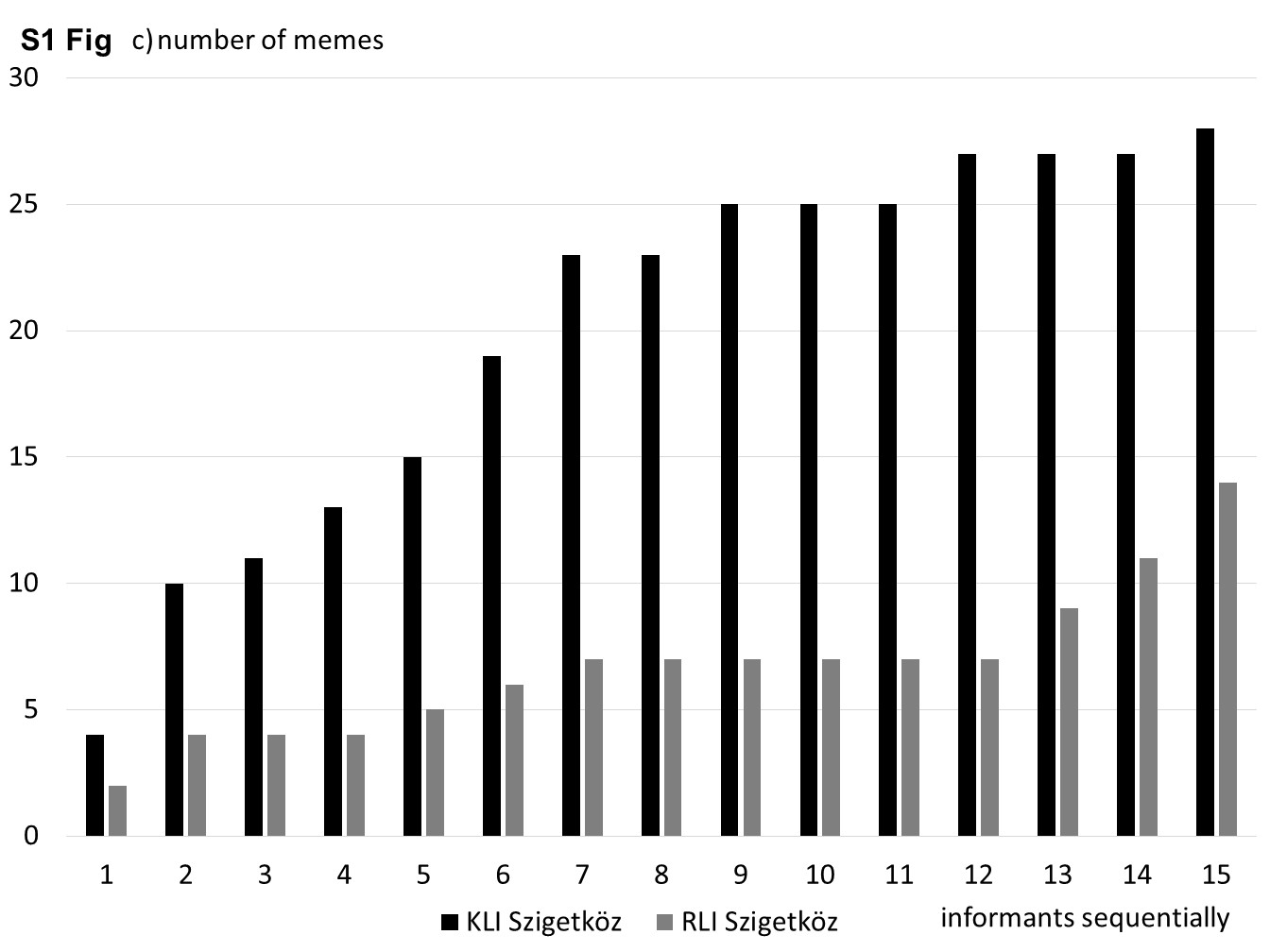

Supplement: S1 Fig — (DOCX) [file pone.0233506.s001.docx]
