# Supplementary material for: Local knowledge about a newly reintroduced, rapidly spreading species (Eurasian beaver) and perception of its impact on ecosystem services
Source: PLoS One. 2020 May 21;15(5):e0233506. doi: 10.1371/journal.pone.0233506 (PMC7241770; doi:10.1371/journal.pone.0233506)
Supplement: S2 Fig — (DOCX) [file pone.0233506.s002.docx]

**S5 Fig.**


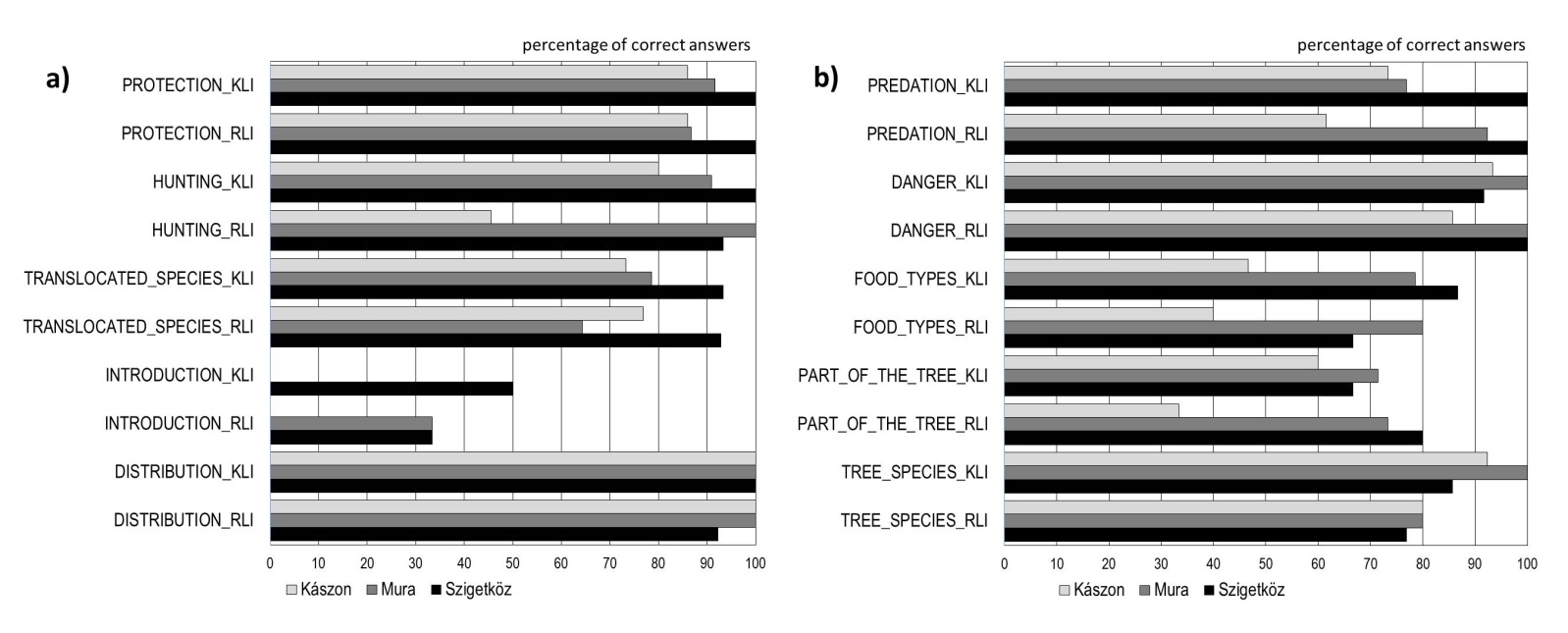


**S5 Fig** Percentage of correct answers related to the key features of beavers (RLI: randomly-selected informants, KLI: local knowledgeable informants). **a)** PROTECTION: are they protected or not?; HUNTING: is it legal to hunt beavers?; TRANSLOCATED SPECIES: how did they arrive?; INTRODUCTION: do you know of any concrete release event?; DISTRIBUTION: where do beavers live in your neighbourhood? **b)** PREDATION: are there any natural predators of beavers?; DANGER: are they dangerous or not to humans?; FOOD TYPES: what do they eat?; PART OF THE TREE: which parts of the tree are eaten by beavers?; TREE SPECIES: which tree species are used by beavers?
