# Supplementary material for: Local knowledge about a newly reintroduced, rapidly spreading species (Eurasian beaver) and perception of its impact on ecosystem services
Source: PLoS One. 2020 May 21;15(5):e0233506. doi: 10.1371/journal.pone.0233506 (PMC7241770; doi:10.1371/journal.pone.0233506)
Supplement: S3 Fig — a) The moving distance of beavers from bodies of water as perceived by the local informants b) The time of beavers’ local reappearance in years, as perceived by the local informants. (DOCX) [file pone.0233506.s003.docx]

**S6 Fig.**


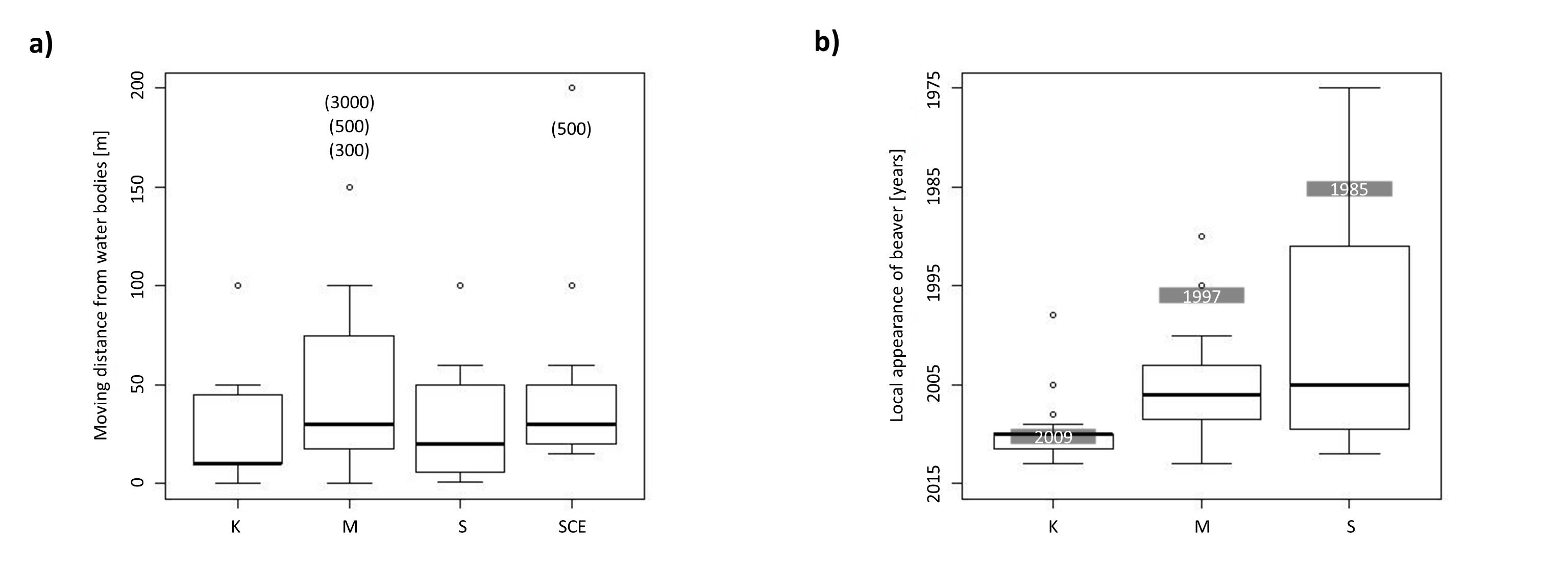


**S6 Fig. a)** The moving distance of beavers from bodies of water as perceived by the local informants (K - Kászon, M - Mura, S - Szigetköz, SCE: values of scientific and conservational experts). **b)** The time of beavers’ local reappearance in years, as perceived by the local informants (three regions: K: Kászon, M: Mura, S: Szigetköz). Numbers indicate the year of reappearance according to the scientific and conservation experts and literature.
