# Supplementary material for: Local knowledge about a newly reintroduced, rapidly spreading species (Eurasian beaver) and perception of its impact on ecosystem services
Source: PLoS One. 2020 May 21;15(5):e0233506. doi: 10.1371/journal.pone.0233506 (PMC7241770; doi:10.1371/journal.pone.0233506)
Supplement: S1 Data — The data sheet used for the interviews. (DOCX) [file pone.0233506.s004.docx]

##### **S2 Questions**

##### The data sheet used for the interviews:

Name of the informant, profession, place, date of birth, date of interview

What does a beaver look like?

What is the size of beavers?

What do they eat?

Which tree species are used by beavers?

Which parts of the tree are eaten by beavers?

How far do beavers go from water bodies?

Are beavers useful or harmful?

What kind of benefits could you mention?

What kind of harms could you mention?

Are there any natural predators of beavers?

Are they dangerous or not to humans?

Is it legal to hunt beavers?

Are they protected or not?

How can you deduce the presence of beavers if you cannot actually see them?

What kind of tree species are used for dams?

Why do beavers build dams?

Which year have the beavers appeared in your neighborhood?

How did they arrive?

Do you know of any concrete release events?

Where do beavers live in your neighborhood?

What is your estimate of local population size?

Is the local population increasing, decreasing or stagnant?

Is this population trend good or bad in your opinion?

What impact do the beavers have on local nature?

What impact do the beavers have on water bodies?

What impact do the beavers have on fish populations?

What impact do the beavers have on riparian woodlands?

What impact do the beavers have on arable fields?

What impact do the beavers have on hay meadows next to the water?

What impact do the beavers have on your life?

What impact do the beavers have on other inhabitants of your village?

Why is it good that the beavers are here?

Who benefits from the beavers being here?
