# Supplementary material for: Local knowledge about a newly reintroduced, rapidly spreading species (Eurasian beaver) and perception of its impact on ecosystem services
Source: PLoS One. 2020 May 21;15(5):e0233506. doi: 10.1371/journal.pone.0233506 (PMC7241770; doi:10.1371/journal.pone.0233506)
Supplement: S1 Table — (DOCX) [file pone.0233506.s005.docx]

##### **S3 Tab.** Some typical examples of the diverse perceptions of beavers related to regulating and cultural services (memes)

# REGULATING: Water regulation

| *they regulate the water flow of streams, and somehow balance water regime* |
| --- |
| *during droughts the level of ground water is raised* |
| *they make reservoirs* |
| *they raise water levels nicely* |
| *they’re good because meadows are flooded, the stream recovers its original bed; bad because marshlands came back* |
| *water level is raised by their dams which is good for the agriculture* [counterbalance drainage] |
| *they don’t build dams, don’t make any difference in waters* |
| *they’re not harmful, just obstructs the water flow* |
| *they clog the stream and the water spreads* |
| *the trees felled into the water course catch a lot of debris, and because of that the stream creates a new bed for itself* |

# REGULATING: Flood protection

| *they moderate floods* |
| --- |
| *rivers shouldn’t be allowed to run wider* |
| *they wash the banks away, this is the damage they cause* |

# REGULATING: Erosion control

| *they stop riverside erosion* [by felling trees and steering water away from the banks] |
| --- |
| *good because they moderate the flow of water, so it doesn’t erode; bad because they chop off trees which would protect the banks* |
| *the water flow is slower, the banks collapse* |
| *they hinder the natural flow of the stream, when water level is high, it cuts in on the sides, and makes wider floodplain* |
| *it has taken a part of our garden* [2 m] |
| *they burrow the banks, make dens; water can cut in more easily and deeply, the current gets it* |
| *the stream have become wider and deeper, and the banks higher* |

# REGULATING: Water purification and water quality regulation

| *water remains pure* |
| --- |
| *dams filter out litter* |
| *the felled trees collect debris* |
| *they purify water* |
| *dams gather debris and sediment* |
| *Water is impounded which means that the amount of sediment increases. That’s surely not good for the water.* |
| *they stir up mud and silt* |
| *they befoul water, slow the flow of water* |

# REGULATING: Lifecycle maintenance, habitat and gene pool protection

| *Well, it’s just that if they build a series of dams, lakes are created. Trout doesn’t like slow water. I can’t tell if fish leave or not, but it’s beneficial for smaller fish, that’s for sure. But as a matter of fact it might be good for trout either, because of the roots, they can hide there.* |
| --- |
| *It’s native. They belong here.* |
| *Well, the impoundments probably help the breeding of fish and that is good for humans. They* [beavers] *provide spawning ground, which is also good from the environmental point of view.* |
| *they help nature to nurse fish by building dams* [so that they can breed and be eaten] |
| *They are good for fish because of the dams. Dams provide shelter. And it’s also positive that new plant and animal species appear.* |
| *God has created beavers with a purpose* |
| *More fish and frogs, and so more storks. They are good for wild boars. Boars can wallow in the ponds where the water is not moving, just impounded.* |
| *they create nesting place for other animals* |
| *maybe nature needs them as well* |
| *grey heron, black stork, and mallards have reappeared* |
| *When water is higher, there are more fish. And they can spawn. They must have a place and function in the food chain.* |
| *the felled trees provide habitat for insects* |
| *it’s a living creature, let it be* |
| *they create habitat for other species like fungi* |
| *they are good for biodiversity* |
| *they are beneficial from the ecological point of view and that is good for everyone* |
| *they provide food for fish: carps eat buds, breams and chub eat the insects from the trees* |
| *the number of fish doesn’t change but they may just help some other animals* |
| *the composition of the forests changes: maples advance* |
| *fish and mallards leave* [to avoid beavers] |
| *They are useless animals and disturb fish. Every creature has a place in nature, but these are simply needless.* |
| *they are bad, because litter is accumulated, and that poisons fish* |
| *trout prefer fast moving waters and beavers stop the rapid flow* |

# CULTURAL: Aesthetic values

| *they clean* [gnaw] *the edges of hay meadows* [the informants consider that as tidiness] |
| --- |
| *it’s good that they clean the banks of the stream* |
| *beautiful, a curiosity* |
| *no lush weeds remain* [good, beavers gnaw plants off] |
| *they clean the groves along the stream, they gnaw off weed trees* |
| [good to have them] *because of the beauty of nature* |
| *they have no impact, but awful to see* |
| *there is quite a chaos on the floodplain* |
| *it takes the filth to the meadows* |
| *stream banks are very ugly* |
| *they fell trees, the landscape becomes ugly* |
| *there is marsh and filth everywhere* |

# CULTURAL: Recreation and ecoturism

| *they block the water flow, so tourists, children and animals can bath* |
| --- |
| *It created a lake. Children could skate in winter.* |
| *nature reserves should be designated* |
| *paddling trips are organized to see the beavers’ lodges and signs of gnawing on the banks of Mura* |
| *tourism can be based on beavers like in the north, visitors can hunt them, it’s extra profit* |
| *it think they wouldn’t attract tourists* |
| *we come here to relax but then sometimes we get upset about them* |
| *it should be perceived as a special attraction, but a station to observe beavers shouldn’t be built* |
| *they have felled the ash tree near my fishing site, they disturb fishing* |
| *they have even gnawed the boats* |
| *the puddles detained after floods are swarming with mosquitos* |
| *one can’t fish for 10-15 minutes, half an hour, not until the water calms down after their movement* |
| *We could bath in the past. Now children are hardly let go* [because beavers make the stream dirty]. |

# CULTURAL: Educational values, information and knowledge

| *they are good for researchers who study them and also for children* |
| --- |
| *it’s good to see them for real* |
| *camps could be organized to show them to children* |
| *there is a beaver-tour in Iklódbördőce* [a trail is blazed] |
| *unconventional biology classes should be organized to 7-8 grade students* |

# CULTURAL: Enjoyment provided by wild species

| *their presence is good for animal lovers, nature conservationists* |
| --- |
| *Well, they are good for children. They love beavers. They have such cute faces. You just can’t dislike them. They are adorable and beautiful animals.* |
| *they are nice animals, I like them, they have a cute face, I disapprove of hurting them* |
| *one more curiosity, now I greet them as friends* |
| *They don’t bother me. I like watching their lodge, what a complex building, and how it raises the water level. They are expert builders, I love their activity. Locals went to admire the lodge: how beautiful, how proficient.* |
| *They build expertly. Even the most proficient lumberjack wouldn’t be able to fell the trees to the same spot.* |
| *They are nice to see, beautiful. They are good because of aesthetic reasons.* |
| *There is a very beautiful tree with signs of gnawing. It’s wonderful.* |
| *Children have enjoyed finding it. The first dams were very much admired. It was a fairy tale, a wonder, magical, …so good. It is a pleasure to see what these little creatures can build.* |
| *They give me half a day of extra work each year, but that’s all. I can live with that… I’m an animal lover, I enjoy having them here.* |
| *it is wonderful how they defend their territories, they are boxing with each other* |
| *I am glad that beavers are here* |
| *I move carefully so that I could observe them better* |
| *sometimes I watch them when I’m in my boat, and I don’t move, I stop* |
| *it could be a Hungarian specialty* |
| *I don’t like them, I am afraid of them* |
| *nobody is happy about them, dams are nice, but it’s not good that we maintain their habitat* |
