# Supplementary material for: Local knowledge about a newly reintroduced, rapidly spreading species (Eurasian beaver) and perception of its impact on ecosystem services
Source: PLoS One. 2020 May 21;15(5):e0233506. doi: 10.1371/journal.pone.0233506 (PMC7241770; doi:10.1371/journal.pone.0233506)
Supplement: S2 Table — (DOCX) [file pone.0233506.s006.docx]

**S4 Tab.** Database of ecosystem services related to the Eurasian beaver in Kászon, Mura and Szigetköz

| **INFORMANT (LANDSCAPE, KNOWLEDGEABLE/RANDOM, Nr.)** | **WATER REGULATION** | **FLOOD PROTECTION** | **DISSERVICE FLOOD** | **EROSION CONTROL** | **WATER PURIFICATION** | **MAINTENANCE OF BIODIVERSITY** | **AESTHETIC** | **RECREATIONAL** | **EDUCATIONAL** | **ENJOYMENT** | **USEFUL OR HARMFUL** | **POPULATION INCREASE GOOD OR NOT** | **IMPACT ON YOUR LIFE** | **IMPACT ON LOCALS** | **IMPACT ON NATURE** |
| --- | --- | --- | --- | --- | --- | --- | --- | --- | --- | --- | --- | --- | --- | --- | --- |
| **K-KLI-1** | 2 | 0 | 0 | 0 | 0 | 0 | 1 | 0 | 0 | 0 | 2 | 0 | 1 | 1 | 4 |
| **K-KLI-2** | 1 | 0 | 0 | 0 | 1 | 1 | 0 | 0 | 1 | 1 | 0 | 0 | 0 | 0 | 2 |
| **K-KLI-3** | 1 | 0 | 0 | 0 | 1 | 3 | 1 | 1 | 0 | 0 | 1 | 0 | 0 | 0 | 2 |
| **K-KLI-4** | 2 | 0 | 0 | 1 | 0 | 2 | 0 | 0 | 1 | 1 | 1 | 0 | 0 | 2 | 2 |
| **K-KLI-5** | 1 | 0 | 0 | 0 | 0 | 2 | 0 | 0 | 0 | 0 | 2 | 0 | 0 | 0 | 2 |
| **K-KLI-6** | 1 | 0 | 0 | 0 | 1 | 0 | 0 | 1 | 0 | 0 | 0 | 0 | 1 | 0 | 3 |
| **K-KLI-7** | 1 | 0 | 0 | 0 | 1 | 1 | 2 | 0 | 0 | 1 | 1 | 0 | 1 | 0 | 3 |
| **K-KLI-8** | 1 | 0 | 0 | 0 | 1 | 0 | 1 | 1 | 1 | 0 | 2 | 0 | 1 | 2 | 2 |
| **K-KLI-9** | 3 | 0 | 0 | 0 | 0 | 1 | 0 | 1 | 0 | 1 | 2 | 0 | 0 | 2 | 2 |
| **K-KLI-10** | 0 | 0 | 0 | 1 | 0 | 1 | 1 | 1 | 0 | 0 | 0 | 0 | 1 | 1 | 2 |
| **K-KLI-11** | 3 | 0 | 0 | 1 | 1 | 1 | 0 | 1 | 1 | 0 | 2 | 0 | 0 | 1 | 1 |
| **K-KLI-12** | 4 | 0 | 1 | 2 | 1 | 0 | 0 | 0 | 0 | 0 | 0 | 0 | 0 | 0 | 1 |
| **K-KLI-13** | 2 | 0 | 1 | 1 | 0 | 0 | 0 | 0 | 0 | 0 | 0 | 0 | 0 | 1 | 2 |
| **K-KLI-14** | 1 | 0 | 0 | 1 | 1 | 0 | 1 | 1 | 0 | 0 | 0 | 1 | 0 | 0 | 3 |
| **K-KLI-15** | 1 | 0 | 0 | 1 | 1 | 2 | 1 | 2 | 0 | 0 | 0 | 2 | 0 | 0 | 1 |
| **K-RLI-1** | 1 | 0 | 3 | 0 | 1 | 0 | 0 | 0 | 0 | 0 | 0 | 0 | 1 | 0 | 2 |
| **K-RLI-2** | 3 | 0 | 0 | 3 | 1 | 3 | 0 | 0 | 0 | 0 | 0 | 1 | 0 | 0 | 3 |
| **K-RLI-3** | 1 | 0 | 0 | 1 | 1 | 0 | 0 | 1 | 0 | 0 | 0 | 0 | 0 | 0 | 0 |
| **K-RLI-4** | 1 | 0 | 0 | 0 | 0 | 0 | 0 | 0 | 0 | 0 | 1 | 0 | 0 | 0 | 2 |
| **K-RLI-5** | 0 | 0 | 0 | 0 | 0 | 0 | 0 | 0 | 0 | 0 | 0 | 0 | 1 | 0 | 2 |
| **K-RLI-6** | 1 | 0 | 0 | 0 | 0 | 1 | 0 | 1 | 0 | 0 | 0 | 0 | 0 | 0 | 1 |
| **K-RLI-7** | 1 | 0 | 0 | 0 | 0 | 0 | 0 | 2 | 0 | 0 | 0 | 1 | 0 | 3 | 2 |
| **K-RLI-8** | 3 | 0 | 0 | 0 | 1 | 1 | 0 | 0 | 0 | 0 | 0 | 0 | 1 | 0 | 3 |
| **K-RLI-9** | 2 | 0 | 0 | 0 | 1 | 1 | 1 | 1 | 0 | 0 | 0 | 0 | 0 | 0 | 2 |
| **K-RLI-10** | 0 | 0 | 0 | 0 | 1 | 2 | 1 | 0 | 0 | 0 | 0 | 0 | 0 | 0 | 2 |
| **K-RLI-11** | 1 | 0 | 1 | 0 | 0 | 1 | 0 | 0 | 0 | 0 | 0 | 0 | 0 | 0 | 2 |
| **K-RLI-12** | 1 | 0 | 0 | 0 | 0 | 0 | 0 | 0 | 0 | 0 | 0 | 0 | 0 | 1 | 2 |
| **K-RLI-13** | 1 | 0 | 0 | 0 | 0 | 0 | 0 | 1 | 0 | 0 | 0 | 0 | 0 | 0 | 2 |
| **K-RLI-14** | 1 | 0 | 0 | 1 | 0 | 1 | 1 | 0 | 0 | 0 | 2 | 0 | 2 | 1 | 0 |
| **K-RLI-15** | 1 | 0 | 0 | 1 | 1 | 0 | 1 | 1 | 0 | 0 | 0 | 1 | 0 | 0 | 3 |
| **INFORMANT (LANDSCAPE, KNOWLEDGEABLE/RANDOM, Nr.)** | **WATER REGULATION** | **FLOOD PROTECTION** | **DISSERVICE FLOOD** | **EROSION CONTROL** | **WATER PURIFICATION** | **MAINTENANCE OF BIODIVERSITY** | **AESTHETIC** | **RECREATIONAL** | **EDUCATIONAL** | **ENJOYMENT** | **USEFUL OR HARMFUL** | **POPULATION INCREASE GOOD OR NOT** | **IMPACT ON YOUR LIFE** | **IMPACT ON LOCALS** | **IMPACT ON NATURE** |
| **M-KLI-1** | 0 | 0 | 0 | 2 | 2 | 1 | 0 | 1 | 0 | 0 | 0 | 0 | 0 | 1 | 1 |
| **M-KLI-2** | 0 | 0 | 0 | 1 | 0 | 0 | 0 | 0 | 0 | 1 | 0 | 0 | 1 | 0 | 1 |
| **M-KLI-3** | 0 | 0 | 0 | 0 | 0 | 0 | 0 | 0 | 0 | 1 | 0 | 0 | 1 | 0 | 1 |
| **M-KLI-4** | 0 | 0 | 0 | 0 | 0 | 0 | 0 | 0 | 0 | 0 | 0 | 0 | 0 | 0 | 1 |
| **M-KLI-5** | 0 | 1 | 0 | 0 | 0 | 0 | 0 | 0 | 1 | 1 | 2 | 0 | 0 | 1 | 0 |
| **M-KLI-6** | 0 | 2 | 0 | 2 | 0 | 0 | 0 | 0 | 0 | 2 | 0 | 0 | 1 | 0 | 0 |
| **M-KLI-7** | 0 | 0 | 0 | 4 | 2 | 1 | 0 | 0 | 1 | 3 | 1 | 0 | 2 | 1 | 2 |
| **M-KLI-8** | 0 | 1 | 0 | 1 | 0 | 0 | 0 | 0 | 0 | 1 | 1 | 1 | 0 | 1 | 1 |
| **M-KLI-9** | 1 | 1 | 0 | 2 | 0 | 0 | 0 | 0 | 0 | 2 | 0 | 0 | 3 | 0 | 3 |
| **M-KLI-10** | 2 | 1 | 0 | 1 | 1 | 0 | 0 | 0 | 0 | 0 | 0 | 0 | 0 | 0 | 1 |
| **M-KLI-11** | 1 | 1 | 0 | 1 | 1 | 0 | 0 | 0 | 1 | 0 | 0 | 0 | 2 | 1 | 1 |
| **M-KLI-12** | 0 | 0 | 0 | 0 | 0 | 0 | 0 | 0 | 0 | 0 | 0 | 0 | 0 | 0 | 0 |
| **M-KLI-13** | 1 | 1 | 1 | 0 | 3 | 0 | 3 | 1 | 1 | 0 | 0 | 1 | 0 | 0 | 0 |
| **M-KLI-14** | 0 | 0 | 0 | 2 | 0 | 0 | 0 | 0 | 0 | 1 | 1 | 1 | 1 | 0 | 1 |
| **M-KLI-15** | 0 | 0 | 0 | 0 | 0 | 0 | 0 | 0 | 0 | 1 | 0 | 0 | 0 | 1 | 0 |
| **M-RLI-1** | 0 | 0 | 0 | 0 | 0 | 0 | 0 | 0 | 0 | 1 | 0 | 0 | 0 | 0 | 0 |
| **M-RLI-2** | 0 | 1 | 0 | 0 | 0 | 0 | 0 | 0 | 0 | 0 | 0 | 0 | 1 | 0 | 1 |
| **M-RLI-3** | 0 | 0 | 0 | 0 | 0 | 0 | 0 | 0 | 0 | 0 | 0 | 0 | 0 | 0 | 1 |
| **M-RLI-4** | 0 | 0 | 0 | 0 | 0 | 0 | 0 | 0 | 0 | 0 | 0 | 0 | 0 | 0 | 0 |
| **M-RLI-5** | 0 | 0 | 0 | 0 | 0 | 0 | 0 | 0 | 0 | 0 | 0 | 0 | 0 | 0 | 2 |
| **M-RLI-6** | 1 | 1 | 0 | 3 | 3 | 0 | 0 | 1 | 1 | 1 | 1 | 0 | 1 | 0 | 2 |
| **M-RLI-7** | 0 | 0 | 0 | 0 | 0 | 0 | 0 | 0 | 0 | 0 | 1 | 0 | 0 | 0 | 1 |
| **M-RLI-8** | 0 | 0 | 0 | 0 | 0 | 0 | 0 | 0 | 0 | 1 | 0 | 0 | 0 | 0 | 0 |
| **M-RLI-9** | 3 | 0 | 0 | 1 | 0 | 0 | 0 | 0 | 0 | 1 | 0 | 1 | 1 | 0 | 0 |
| **M-RLI-10** | 1 | 1 | 0 | 0 | 1 | 0 | 1 | 0 | 0 | 1 | 1 | 1 | 0 | 0 | 3 |
| **M-RLI-11** | 1 | 0 | 0 | 1 | 1 | 0 | 0 | 0 | 0 | 0 | 0 | 1 | 1 | 0 | 4 |
| **M-RLI-12** | 0 | 0 | 0 | 1 | 0 | 0 | 0 | 0 | 0 | 1 | 2 | 1 | 1 | 0 | 0 |
| **M-RLI-13** | 1 | 0 | 0 | 1 | 0 | 0 | 0 | 0 | 0 | 0 | 1 | 1 | 2 | 1 | 2 |
| **M-RLI-14** | 2 | 1 | 0 | 0 | 0 | 0 | 0 | 0 | 0 | 1 | 0 | 0 | 0 | 0 | 0 |
| **M-RLI-15** | 0 | 1 | 0 | 1 | 2 | 0 | 0 | 1 | 0 | 3 | 0 | 0 | 2 | 1 | 3 |
| **INFORMANT (LANDSCAPE, KNOWLEDGEABLE/RANDOM, Nr.)** | **WATER REGULATION** | **FLOOD PROTECTION** | **DISSERVICE FLOOD** | **EROSION CONTROL** | **WATER PURIFICATION** | **MAINTENANCE OF BIODIVERSITY** | **AESTHETIC** | **RECREATIONAL** | **EDUCATIONAL** | **ENJOYMENT** | **USEFUL OR HARMFUL** | **POPULATION INCREASE GOOD OR NOT** | **IMPACT ON YOUR LIFE** | **IMPACT ON LOCALS** | **IMPACT ON NATURE** |
| **S-KLI-1** | 0 | 0 | 0 | 1 | 0 | 3 | 0 | 0 | 0 | 0 | 0 | 0 | 0 | 0 | 2 |
| **S-KLI-2** | 1 | 0 | 0 | 2 | 0 | 3 | 0 | 1 | 0 | 1 | 2 | 0 | 2 | 2 | 2 |
| **S-KLI-3** | 0 | 0 | 0 | 0 | 0 | 2 | 0 | 0 | 0 | 1 | 0 | 1 | 1 | 0 | 2 |
| **S-KLI-4** | 0 | 0 | 1 | 0 | 0 | 1 | 0 | 1 | 0 | 1 | 0 | 1 | 1 | 0 | 1 |
| **S-KLI-5** | 0 | 0 | 0 | 0 | 0 | 1 | 0 | 0 | 0 | 0 | 0 | 0 | 0 | 0 | 0 |
| **S-KLI-6** | 0 | 0 | 0 | 0 | 1 | 3 | 0 | 1 | 0 | 0 | 0 | 1 | 1 | 2 | 3 |
| **S-KLI-7** | 0 | 0 | 0 | 0 | 1 | 1 | 0 | 2 | 0 | 0 | 0 | 0 | 1 | 0 | 2 |
| **S-KLI-8** | 0 | 0 | 0 | 0 | 0 | 1 | 0 | 0 | 0 | 0 | 0 | 0 | 0 | 0 | 1 |
| **S-KLI-9** | 0 | 0 | 0 | 0 | 0 | 4 | 1 | 1 | 0 | 1 | 2 | 1 | 0 | 1 | 1 |
| **S-KLI-10** | 0 | 0 | 0 | 0 | 0 | 1 | 1 | 0 | 0 | 1 | 0 | 1 | 2 | 0 | 0 |
| **S-KLI-11** | 0 | 0 | 0 | 0 | 0 | 0 | 0 | 0 | 0 | 0 | 0 | 0 | 1 | 0 | 0 |
| **S-KLI-12** | 0 | 0 | 0 | 0 | 0 | 0 | 0 | 1 | 0 | 0 | 0 | 0 | 0 | 1 | 1 |
| **S-KLI-13** | 1 | 0 | 0 | 0 | 0 | 3 | 0 | 0 | 0 | 0 | 0 | 1 | 0 | 1 | 2 |
| **S-KLI-14** | 0 | 0 | 0 | 0 | 0 | 1 | 0 | 0 | 0 | 0 | 0 | 0 | 0 | 1 | 0 |
| **S-KLI-15** | 0 | 0 | 0 | 0 | 0 | 0 | 1 | 2 | 0 | 1 | 1 | 1 | 1 | 1 | 2 |
| **S-RLI-1** | 0 | 0 | 0 | 0 | 0 | 1 | 0 | 0 | 0 | 1 | 0 | 1 | 1 | 1 | 1 |
| **S-RLI-2** | 0 | 0 | 0 | 0 | 0 | 0 | 0 | 2 | 0 | 0 | 0 | 0 | 0 | 0 | 0 |
| **S-RLI-3** | 0 | 0 | 0 | 0 | 0 | 0 | 0 | 0 | 0 | 0 | 0 | 1 | 0 | 0 | 0 |
| **S-RLI-4** | 0 | 0 | 0 | 0 | 0 | 0 | 0 | 0 | 0 | 0 | 0 | 0 | 0 | 0 | 0 |
| **S-RLI-5** | 0 | 0 | 0 | 0 | 0 | 1 | 0 | 0 | 0 | 0 | 0 | 0 | 0 | 0 | 0 |
| **S-RLI-6** | 1 | 0 | 1 | 0 | 0 | 1 | 0 | 1 | 0 | 0 | 0 | 0 | 0 | 0 | 0 |
| **S-RLI-7** | 0 | 0 | 0 | 0 | 0 | 2 | 0 | 0 | 0 | 0 | 0 | 0 | 0 | 2 | 0 |
| **S-RLI-8** | 0 | 0 | 0 | 0 | 0 | 0 | 0 | 0 | 0 | 0 | 0 | 0 | 0 | 0 | 0 |
| **S-RLI-9** | 0 | 0 | 0 | 0 | 0 | 0 | 0 | 0 | 0 | 0 | 0 | 0 | 0 | 0 | 0 |
| **S-RLI-10** | 0 | 0 | 0 | 0 | 0 | 1 | 0 | 0 | 0 | 0 | 0 | 1 | 0 | 0 | 1 |
| **S-RLI-11** | 0 | 0 | 0 | 0 | 0 | 0 | 0 | 0 | 0 | 0 | 0 | 0 | 0 | 0 | 0 |
| **S-RLI-12** | 0 | 0 | 0 | 0 | 0 | 1 | 0 | 0 | 0 | 0 | 0 | 0 | 1 | 1 | 2 |
| **S-RLI-13** | 0 | 0 | 0 | 0 | 0 | 1 | 1 | 0 | 0 | 0 | 0 | 1 | 1 | 0 | 2 |
| **S-RLI-14** | 0 | 0 | 0 | 0 | 0 | 0 | 1 | 0 | 0 | 0 | 0 | 0 | 0 | 0 | 2 |
| **S-RLI-15** | 1 | 0 | 0 | 0 | 1 | 1 | 2 | 1 | 0 | 0 | 0 | 1 | 1 | 1 | 2 |

|  |  | **number of informants with negative neutral or positive perception** | | | | | |
| --- | --- | --- | --- | --- | --- | --- | --- |
|  |  | **negative** | **neutral** | **positive** | **does not know** | **no data** | **not relevant** |
| **K-KLI** | **CULTIVATED CROPS** | 2 | 11 | 0 | 2 | 0 | 0 |
| **K-KLI** | **FISH** | 3 | 8 | 1 | 2 | 1 | 0 |
| **K-KLI** | **TIMBER** | 0 | 2 | 0 | 1 | 1 | 11 |
| **K-KLI** | **FOOD (INCL. HAY)** | 3 | 9 | 0 | 2 | 1 | 0 |
| **K-KLI** | **FUEL** | 2 | 6 | 2 | 0 | 5 | 0 |
| **K-KLI** | **OTHER PROVISIONING** | 8 | 6 | 0 | 1 | 0 | 0 |
| **K-RLI** | **CULTIVATED CROPS** | 0 | 9 | 0 | 2 | 4 | 0 |
| **K-RLI** | **FISH** | 10 | 1 | 0 | 2 | 2 | 0 |
| **K-RLI** | **TIMBER** | 0 | 1 | 0 | 0 | 0 | 14 |
| **K-RLI** | **FOOD (INCL. HAY)** | 5 | 6 | 1 | 2 | 1 | 0 |
| **K-RLI** | **FUEL** | 0 | 4 | 2 | 0 | 9 | 0 |
| **K-RLI** | **OTHER PROVISIONING** | 11 | 3 | 1 | 0 | 0 | 0 |
| **M-KLI** | **CULTIVATED CROPS** | 7 | 6 | 0 | 0 | 2 | 0 |
| **M-KLI** | **FISH** | 1 | 14 | 0 | 0 | 0 | 0 |
| **M-KLI** | **TIMBER** | 1 | 14 | 0 | 0 | 0 | 0 |
| **M-KLI** | **FOOD (INCL. HAY)** | 1 | 11 | 1 | 0 | 1 | 1 |
| **M-KLI** | **FUEL** | 0 | 15 | 0 | 0 | 0 | 0 |
| **M-KLI** | **OTHER PROVISIONING** | 10 | 5 | 0 | 0 | 0 | 0 |
| **M-RLI** | **CULTIVATED CROPS** | 10 | 5 | 0 | 0 | 0 | 0 |
| **M-RLI** | **FISH** | 1 | 13 | 0 | 1 | 0 | 0 |
| **M-RLI** | **TIMBER** | 0 | 13 | 0 | 0 | 2 | 0 |
| **M-RLI** | **FOOD (INCL. HAY)** | 2 | 12 | 0 | 0 | 1 | 0 |
| **M-RLI** | **FUEL** | 0 | 15 | 0 | 0 | 0 | 0 |
| **M-RLI** | **OTHER PROVISIONING** | 9 | 6 | 0 | 0 | 0 | 0 |
| **S-KLI** | **CULTIVATED CROPS** | 4 | 11 | 0 | 0 | 0 | 0 |
| **S-KLI** | **FISH** | 4 | 7 | 0 | 0 | 3 | 1 |
| **S-KLI** | **TIMBER** | 12 | 2 | 0 | 1 | 0 | 0 |
| **S-KLI** | **FOOD (INCL. HAY)** | 0 | 11 | 1 | 1 | 2 | 0 |
| **S-KLI** | **FUEL** | 10 | 2 | 1 | 0 | 2 | 0 |
| **S-KLI** | **OTHER PROVISIONING** | 10 | 3 | 0 | 0 | 2 | 0 |
| **S-RLI** | **CULTIVATED CROPS** | 2 | 10 | 0 | 2 | 0 | 1 |
| **S-RLI** | **FISH** | 5 | 9 | 0 | 1 | 0 | 0 |
| **S-RLI** | **TIMBER** | 13 | 0 | 0 | 0 | 2 | 0 |
| **S-RLI** | **FOOD (INCL. HAY)** | 0 | 13 | 0 | 0 | 2 | 0 |
| **S-RLI** | **FUEL** | 12 | 1 | 0 | 0 | 2 | 0 |
| **S-RLI** | **OTHER PROVISIONING** | 12 | 1 | 0 | 0 | 2 | 0 |
